# Star Lightweight Convolution and NDT-RRT: An Integrated Path Planning Method for Walnut Harvesting Robots

**DOI:** 10.3390/s26010305

**Published:** 2026-01-02

**Authors:** Xiangdong Liu, Xuan Li, Bangbang Chen, Jijing Lin, Kejia Zhuang, Baojian Ma

**Affiliations:** 1 School of Mechatronic Engineering, Xinjiang Institute of Technology, Aksu 843100, China; 2017011@xjit.edu.cn (X.L.); 2018017@xjit.edu.cn (X.L.); 2023274@xjit.edu.cn (J.L.); 11813019@zju.edu.cn (B.M.); 2 School of Mechanical Electronic Engineering, Wuhan University of Technology, Wuhan 430070, China; zhuangkj@whut.edu.cn

**Keywords:** fallen walnut, robot picking, target detection, path planning, model deployment

## Abstract

To address issues such as slow response speed and low detection accuracy in fallen walnut picking robots in complex orchard environments, this paper proposes a detection and path planning method that integrates star-shaped lightweight convolution with NDT-RRT. The method includes the improved lightweight detection model YOLO-FW and an efficient path planning algorithm NDT-RRT. YOLO-FW enhances feature extraction by integrating star-shaped convolution (Star Blocks) and the C3K2 module in the backbone network, while the introduction of a multi-level scale pyramid structure (CA_HSFPN) in the neck network improves multi-scale feature fusion. Additionally, the loss function is replaced with the PIoU loss, which incorporates the concept of Inner-IoU, thus improving regression accuracy while maintaining the model’s lightweight nature. The NDT-RRT path planning algorithm builds upon the RRT algorithm by employing node rejection strategies, dynamic step-size adjustment, and target-bias sampling, which reduces planning time while maintaining path quality. Experiments show that, compared to the baseline model, the YOLO-FW model achieves precision, recall, and mAP@0.5 of 90.6%, 90.4%, and 95.7%, respectively, with a volume of only 3.62 MB and a 30.65% reduction in the number of parameters. The NDT-RRT algorithm reduces search time by 87.71% under conditions of relatively optimal paths. Furthermore, a detection and planning system was developed based on the PySide6 framework on an NVIDIA Jetson Xavier NX embedded device. On-site testing demonstrated that the system exhibits good robustness, high precision, and real-time performance in real orchard environments, providing an effective technological reference for the intelligent operation of fallen walnut picking robots.

## 1. Introduction

Walnut, an important tree species in the Juglandaceae family, is not only a traditional medicinal and edible crop in China but is also regarded as one of the “four major nuts” in the world, alongside hazelnuts, almonds, and cashews. Walnuts are highly valued for their numerous active components, including proteins, vitamins, and minerals, which offer a variety of benefits such as improving nerve conduction, enhancing cardiovascular function, and possessing antioxidant properties. In recent years, walnuts have gained increasing popularity among consumers [1,2]. With the growing demand for walnuts, the scale of walnut cultivation continues to expand. According to statistics, by 2025, the planting area of walnuts in China is expected to exceed 497,600 hectares, with an annual production surpassing 5.935 million tons. However, the current walnut industry remains heavily reliant on manual labor during the harvest process, with low levels of mechanization and automation, severely limiting the industry’s sustainable development. Therefore, achieving automated harvesting is key to advancing the modernization of the walnut industry.

With the rapid development of artificial intelligence and robotics, agricultural robots for walnut picking have emerged as an effective solution to the current challenges. For instance, a collection device designed by Yang Shuhua et al. [3], which combines a robotic arm with an end-effector for coordinated operation, requires manual guidance for positioning, rendering it inefficient for practical orchard tasks and failing to achieve full automation. The primary challenge lies in the fact that fallen walnuts in non-structured orchards are obscured by branches and leaves, subject to variable lighting, and often blend into backgrounds, making it difficult for robots to perform precise and rapid operations in visual perception and decision-making control. Thus, developing high-precision recognition and rapid-response robot path planning methods is the core challenge for achieving automated walnut harvesting.

In recent years, deep learning techniques have made significant progress in image object detection, segmentation, and recognition, and have been widely applied in fruit recognition tasks [4,5]. Compared to traditional machine vision methods that rely on manual features such as color and texture [6,7], deep learning excels in extracting rich and deep image features, significantly enhancing detection accuracy and robustness. Single-stage detection networks such as YOLO and SSD, which divide images into grids and directly predict objects, balance both detection speed and accuracy and are widely used in agricultural scenarios. Although research on fallen walnut recognition is relatively scarce, significant breakthroughs have been made in similar fruit research. For instance, to enhance real-time performance for specific tasks, Shi Yi et al. [8] adopted a transfer learning strategy to improve the YOLOv8 model’s ability to identify young apples and introduced an ECA module to maintain lightness while achieving a mAP50 of 97.3%. Liu Yucheng et al. [9] embedded an SPDConv module into YOLOv8 to enhance small object detection capabilities, combined with an EMA attention mechanism and Wise-IoU loss function, improving lemon detection mAP to 92.4%. Kangkang Qi et al. [10] introduced a state-space model to reduce computational load and designed LS Block and RG Block dual modules to improve the robustness of the model, achieving more than 96% detection accuracy for different maturity levels of shiitake mushrooms. Despite these successful results in specific tasks, challenges remain in orchard environments where background interference, dense targets, or occlusions are present, leading to issues such as insufficient detection accuracy and poor robustness. Furthermore, current research on fallen walnuts mainly focuses on recognition, with few studies addressing the efficient path planning for robot picking after object recognition.

Regarding path planning, mainstream algorithms can be divided into graph search methods [11,12,13] (such as A, Dijkstra) and random sampling methods [14,15] (such as RRT, RRT). Although graph search algorithms ensure path connectivity, their computational complexity increases sharply with environmental complexity, making them difficult to meet real-time requirements. In contrast, RRT-based algorithms, due to their random sampling mechanism, demonstrate stronger adaptability and search efficiency in high-dimensional and complex environments, with simpler algorithm structures and lower computational costs, making them more suitable for path planning tasks in orchard environments. However, traditional RRT algorithms still suffer from issues such as blind sampling, slow convergence, and suboptimal paths. To address these limitations, researchers have proposed various improvements, such as Kuffner et al. [16] who significantly improved planning efficiency by constructing bidirectional search trees; Klemm et al. [17] who combined RRT* and RRT-Connect advantages to propose an asymptotically optimal random motion planning algorithm, effectively reducing runtime; Chen et al. [18] who introduced a third-node strategy into RRT-Connect and used a quadtree structure for greedy expansion to further improve search efficiency; Chen Xiaolong et al. [19] who integrated artificial potential field guidance and dynamic step size strategies into RRT*, reducing the average planning time by 50.88% and shortening path lengths by 6.24% through path pruning and smoothing mechanisms; Zheng Zebin et al. [20] who introduced an adaptive target bias strategy and dynamic probability node rejection mechanism into the Informed RRT* framework, reducing search time by 97.36% and increasing success rate by 283.33%; and Zhang Yanjun et al. [21] who enhanced search efficiency by 81.98% using adaptive step size and AdaGrad optimization methods with cubic B-spline path optimization in the P-RRT* framework. These path planning methods have greatly improved search efficiency and path quality, providing valuable insights for the path planning research of fallen walnut harvesting robots in non-structured environments. This paper proposes a method combining a star-shaped lightweight convolution deep learning recognition model with the dynamic enhanced fast-expanding random tree (NDT-RRT) path planning approach.

**Figure 1:**
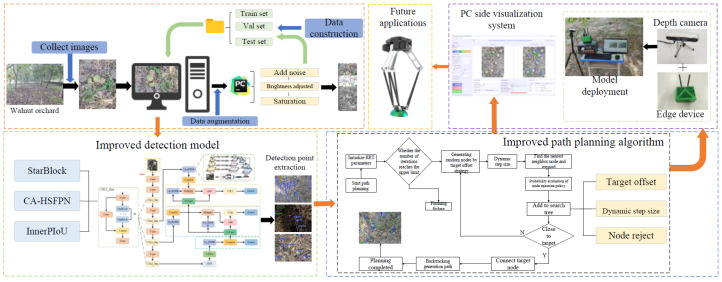
Research framework diagram.

In conclusion, to address the challenges of inadequate detection model accuracy, poor real-time performance, and limited path planning efficiency in fallen walnut picking robots, this study proposes a path planning method integrating star-shaped lightweight convolution with NDT-RRT. As shown in Figure 1, this research first constructs a fallen walnut dataset in a non-structured orchard environment for training the designed lightweight detection model. Then, the detection results are used as prior information to drive the improved NDT-RRT algorithm for efficient path planning. Finally, the entire system is deployed on edge computing devices and tested in an orchard, verifying its feasibility and robustness in real-world scenarios. The main contributions of this study are as follows:

(1) In the fallen walnut detection model, a lightweight Star Blocks convolution and a multi-level scale pyramid feature network (CA_HSFPN) are proposed to optimize the C3K2 module in the original deep learning model, effectively enhancing the fusion of high-dimensional non-linear features and the recognition accuracy of walnuts under multi-scale targets. Additionally, an Inner_PIoU-based loss function is introduced to accelerate model convergence and improve localization accuracy.

(2) In the path planning algorithm, an improved NDT-RRT algorithm is proposed, which significantly enhances path search efficiency and final path quality by introducing node rejection strategies, dynamic step size mechanisms, and target bias sampling strategies.

(3) An integrated detection and planning visualization system is developed based on the Pyside6 framework and successfully deployed on the NVIDIA Jetson Xavier NX edge device. Test results in real orchard environments demonstrate that the proposed path planning method for the picking robot has good feasibility and superior overall performance.

## 2. Materials and Methods

### 2.1. Fallen Walnuts Dataset Construction

The dataset for this study was collected at the Walnut Forest Farm in Wensu County, Aksu, Xinjiang. A Huawei Nova7 smartphone was used as the data acquisition device. Between 10 and 15 September 2025, images were captured for two major walnut varieties, “Wen185” and “Xin2.” To comprehensively reflect the true distribution characteristics of Fallen Walnuts in a natural, unstructured orchard environment, the data collection process systematically covered a variety of real-world conditions, including different light intensities (low light, bright light), camera angles (horizontal, inclined), walnut surface states (with green skin, without green skin), and shooting distances (0.2–1.5 m). A total of 2054 raw images were collected and stored in JPEG format.

**Figure 2:**
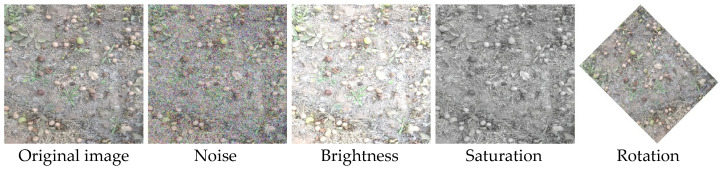
Data augmentation of the fallen walnuts dataset.

Given that visual systems in real orchard operations are often interfered with by multiple environmental factors, and to enhance the dataset’s generalization ability and model robustness, data augmentation was performed on the raw images using Python. The specific augmentation techniques included random noise (65–80), brightness adjustment (brightness factor 1.3), saturation variation (saturation factor 0.01), and random rotation transformation (clockwise rotation of 45°). Some examples of these augmentations are shown in Figure 2. After augmentation, the total number of images was expanded to 4600, which were split into training (3220 images), validation (920 images), and testing (460 images) datasets in a 7:2:1 ratio for model training, hyperparameter tuning, and final performance evaluation, respectively. During the annotation phase, the Labelimg software was used to label pickable walnuts as “Pickable,” while non-pickable targets were left unannotated.

### 2.2. Lightweight Fallen Walnuts Detection Algorithm

**Figure 3:**
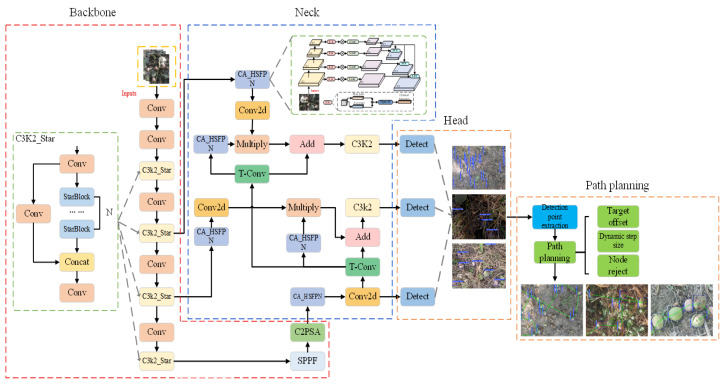
YOLO-FW model architecture.

To achieve fast and accurate detection of fallen walnuts in the complex orchard environment and to provide a reliable basis for subsequent picking path planning, this study proposes a real-time lightweight fallen walnuts detection model, YOLO-FW (Fallen Walnuts), based on the popular deep learning model YOLOv11n. The model structure is shown in Figure 3. In the backbone network, the original C3K2 module was improved by integrating the Star Blocks convolution structure. In the neck network, a multi-scale feature pyramid network (CA_HSFPN) was introduced to replace the original C3K2 module. In the loss function, the original Complete IoU (CIoU) loss was substituted with the enhanced Powerful IoU (PIoU) loss, which was further optimized by drawing inspiration from the concept of Inner-IoU, thereby improving the bounding box regression process.

#### 2.2.1. StarNet Feature Extraction Structure

**Figure 4:**
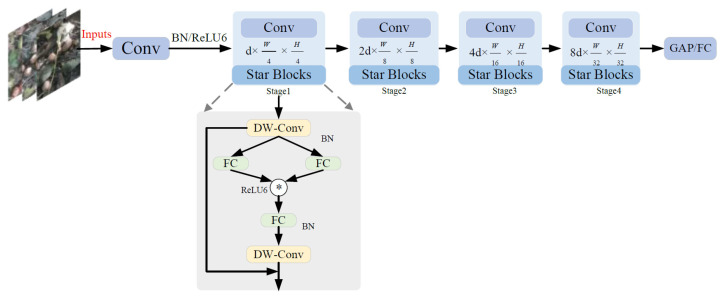
StarNet network architecture.

The C3K2 feature extraction module, a core component of YOLOv11n, primarily extracts image features from different scales, obtaining multi-scale information through parallel convolutional branches and combining shallow texture features with deep semantic features. This allows the detection head to receive richer feature representations [22]. However, the dual fusion mechanism involved requires multiple operations on feature maps, creating a bottleneck between computational efficiency and performance on resource-constrained embedded devices. To address this issue, this study incorporates the Star Block from StarNet [23] to refine the C3K2 module, introducing it into one branch of the dual-branch feature extraction for feature transformation, while keeping the other branch unchanged. The Star Block can implicitly map input features to a high-dimensional nonlinear space, extracting more comprehensive feature information while maintaining low latency and excellent representation capabilities. Its structure is shown in Figure 4.

In the StarNet network architecture, Star Blocks serve as the core component. They use element-wise multiplication to implicitly map the input features to a high-dimensional nonlinear space, enabling efficient feature interaction. In a single-layer neural network structure, this operation can be expressed as the element-wise multiplication of features after two linear transformations, i.e., $\left(W_{1}^{T}X+B_{1})*(W_{2}^{T}X+B_{2}\right)$. To simplify, the weight matrix and bias term are combined as a single parameter matrix W = [W; B], and the input is extended as X = [X; 1], resulting in the standard form of the star operation as ${(W}_{1}^{T}{X)*(W}_{2}^{T}X)$.

Under the setting of a single output channel and a single input element, the parameters $w_{1},w_{2}$ and the input *x* are defined with dimensions (*d* + 1) × (*d*′ + 1), where d is the number of input channels. Furthermore, to adapt to multi-output channels and multi-element input scenarios, $W_{1},W_{2}$ are expanded to dimensions (*d* + 1) × (*d*′ + 1), and the input X is expanded to (*d* + 1) × *n*. Based on the above representation, the star operation can be expressed as shown in Equation (1).

(1) $$\begin{array}{ll} w_{1}^{T}x*w_{2}^{T}x & =\left(\sum_{i=1}^{d+1}w_{1}^{i}x^{i}\right)*\left(\sum_{j=1}^{d+1}w_{2}^{j}x^{j}\right)\\ & =\sum_{i=1}^{d+1}\sum_{j=1}^{d+1}w_{1}^{i}w_{2}^{j}x^{i}x^{j}\\ & =\underset{(d+2)(d+1)/2}{\underset{︸}{\alpha_{\left(1,1\right)}x^{1}x^{1}+\cdots +\alpha_{\left(4,5\right)}x^{4}x^{5}+\cdots +\alpha_{\left(d+1,d+1\right)}x^{d+1}x^{d+1}}} \end{array}$$

In this equation, i,j are the index channels, and α represents each coefficient as:

(2) $$\alpha (i,j)=\left\{\begin{array}{l} w_{1}^{i}w_{2}^{j}\;\;\;\;\;\;\;\;\;\;\;\;\;\;\;\mathrm{if}\;i==j\\ w_{1}^{i}w_{2}^{j}+w_{1}^{j}w_{2}^{i}\;\mathrm{if}\;i!=j \end{array}\right.$$

**Figure 5:**
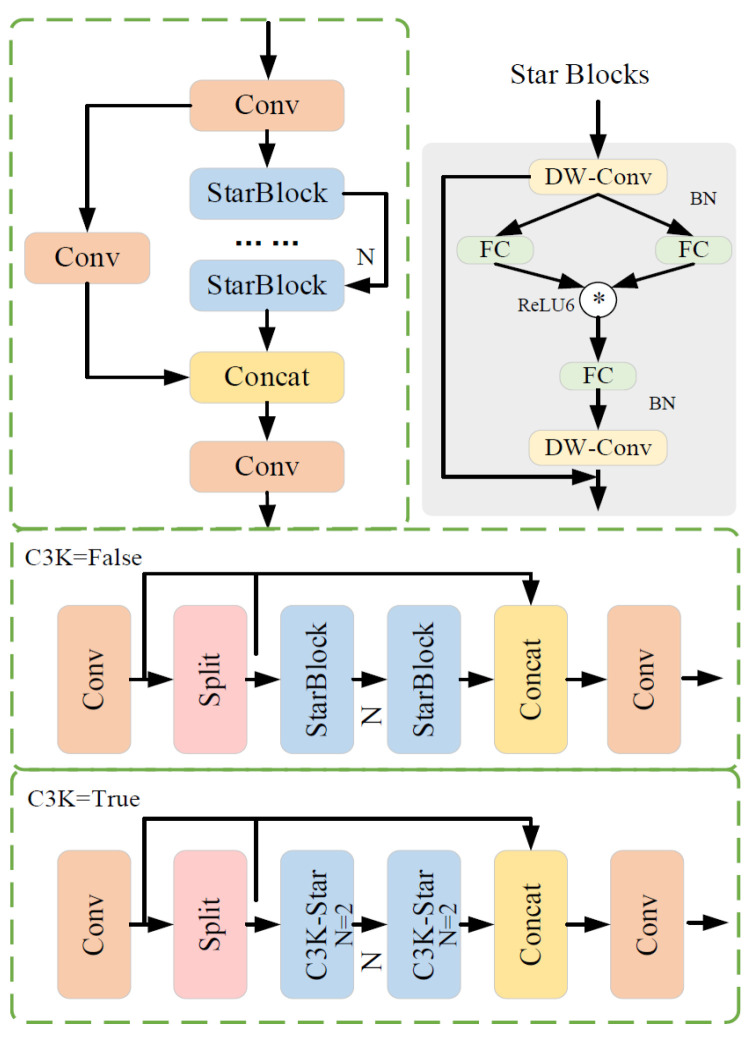
C3K2-Star network architecture.

In the rewritten star operation, it can be expanded into a combination of (*d* + 2)(*d* + 1)/2 distinct terms, as shown in Equation (2). Except for the part related to the bias term $\alpha_{\left(d+1,:\right)}x^{\left(d+1\right)}x$, which is a linear term, all other terms exhibit a nonlinear relationship with the input *x*, indicating that they are independent and implicitly encoded function mappings. Therefore, while the star operation is performed in a d-dimensional space, it achieves representation in a high-dimensional implicit feature space of ${\left(d/\sqrt{2}\right)}^{2}$, significantly enhancing feature dimensionality and expressive power without increasing additional memory overhead. The improved C3K2-Star network structure is shown in Figure 5.

#### 2.2.2. CA_HSFPN Multi-Level Scale Feature Network Structure

**Figure 6:**
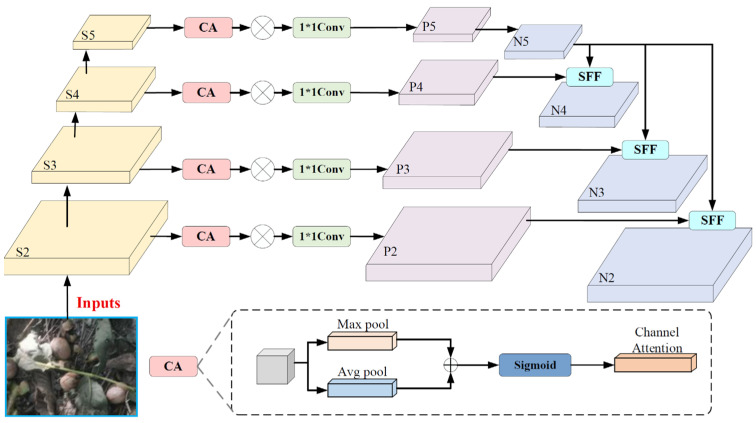
CA_HSFPN network architecture.

To enhance the performance of the neck network in multi-scale feature extraction and fusion, and thereby improve the accuracy and efficiency of object detection, this study introduces the CA_HSFPN module [24,25], which is a secondary improvement based on the multi-level feature pyramid network. This structure consists of two main parts: feature selection and feature fusion, as shown in Figure 6.

In the feature selection phase, the Coordinate Attention (CA) mechanism is introduced to replace the original channel attention mechanism. The CA mechanism not only models the inter-channel dependencies but also explicitly incorporates positional information, enabling bidirectional position encoding that captures both cross-channel and directional awareness. This allows the model to more precisely focus on the contextual information of the target space. During the embedding of the coordinate information, CA uses pooling kernels of sizes (*H*, 1) and (1, *W*) to encode each channel along the height and width directions, respectively. The output for the *c*-th channel along the height *H* is given by Equation (3), and the output along the width *W* is provided by Equation (4). In the coordinate attention generation phase, the positional information in the horizontal and vertical directions is concatenated and processed through a 1 × 1 convolutional transformation function to generate attention weight tensors, *g^h^* and *g^w^*, with the same number of channels, as shown in Equations (5) and (6). Finally, these two tensors are multiplied together to generate the coordinate attention weights, as shown in Equation (7).

(3) $$z_{c}^{h}(h)=\frac{1}{W}\sum_{0\leq i\leq w}x_{c}(h,j)$$

(4) $$z_{c}^{w}(w)=\frac{1}{H}\sum_{0\leq j\leq w}x_{c}(i,w)$$

(5) $$g^{h}=\sigma (F_{h}(f^{h}))$$

(6) $$g^{w}=\sigma (F_{w}(f^{w}))$$

(7) $$y_{n}(i,j)=x_{n}(i,j)\times g_{n}^{h}(i)\times g_{n}^{w}(j)$$

In the equations, *H* refers to the height of the input feature map, *W* refers to the width of the input feature map, *x_c_*(*h*, *j*) represents the input along the horizontal direction, *x_c_*(*i*, *w*) represents the input along the vertical direction, σ is the sigmoid function, $y_{n}(i,j)$ denotes the output of the *n*-th channel, $x_{n}(i,j)$ represents the input of the *n*-th channel, $g_{n}^{h}(i,j)$ refers to the weight along the horizontal direction of the *n*-th channel, and $g_{n}^{w}(i,j)$ refers to the weight along the vertical direction of the *n*-th channel.

#### 2.2.3. Loss Function Optimization

To enhance the accuracy and efficiency of bounding box regression, this study introduces an improvement to the original CIoU loss function in YOLOv11n. Although CIoU is widely used to optimize the discrepancy between predicted and ground truth boxes, it lacks sensitivity to subtle changes in bounding box details when calculating the overlap area, thus limiting the potential for further improvements in detection accuracy. To address this issue, this paper incorporates the PIoU loss function and the concept of InnerIoU [26,27], and proposes a novel Inner_PIoU loss function, as shown in Equations (8)–(16). The principle behind this approach lies in the use of auxiliary bounding boxes and the introduction of a scaling factor, enabling flexible control over the loss computation area. This allows for adaptive gradient distribution and regression prioritization within different IoU intervals, effectively enhancing the model’s sensitivity to small variations in bounding boxes and improving localization accuracy.

(8) $$b_{l}^{gt}=x_{c}^{gt}-\frac{\omega^{gt}*ratio}{2},b_{r}^{gt}=x_{c}^{gt}+\frac{\omega^{gt}*ratio}{2}$$

(9) $$b_{t}^{gt}=y_{c}^{gt}-\frac{h^{gt}*ratio}{2},b_{b}^{gt}=y_{c}^{gt}+\frac{h^{gt}*ratio}{2}$$

(10) $$b_{l}=x_{c}-\frac{\omega *ratio}{2},b_{r}=x_{c}+\frac{\omega *ratio}{2}$$

(11) $$b_{t}=y_{c}-\frac{h*ratio}{2},b_{b}=y_{c}+\frac{h*ratio}{2}$$

(12) $$L_{PIoU}=1-PIoU=L_{IoU}+f(P),0\leq L_{PIoU}\leq 2$$

(13) $$inter=(\min(b_{r}^{gt},b_{r})-\max(b_{l}^{gt},b_{l}))*(\min(b_{b}^{gt},b_{b})-\max(b_{t}^{gt},b_{t}))$$

(14) $$union=(\omega^{gt}*h^{gt})*{\left(ratio\right)}^{2}+(\omega *h)*{\left(ratio\right)}^{2}-inter$$

(15) $$IoU^{inner}=\frac{inter}{union}$$

(16) $$L_{Inner\_PIoU}=L_{PIoU}+IoU+IoU^{inner}$$

where *P* is the penalty factor in IoU; h and ω represent the height and width of the input image, respectively; $h^{gt}$ and $\omega^{gt}$ denote the height and width of the ground-truth box, respectively; *ratio* stands for the scaling factor, usually set within [0.5, 1.5]; *inter* indicates the area of intersection between the auxiliary bounding boxes; $x_{c}^{gt}$ and $y_{c}^{gt}$ represent the center points of the ground-truth box and the inner ground-truth box, respectively; $x_{c}$ and $y_{c}$ denote the center points of the anchor box and the inner anchor box, respectively; and IoU refers to the intersection over union between the predicted box and the ground-truth box.

### 2.3. Fallen Walnuts Pickup Path Planning Algorithm

**Figure 7:**
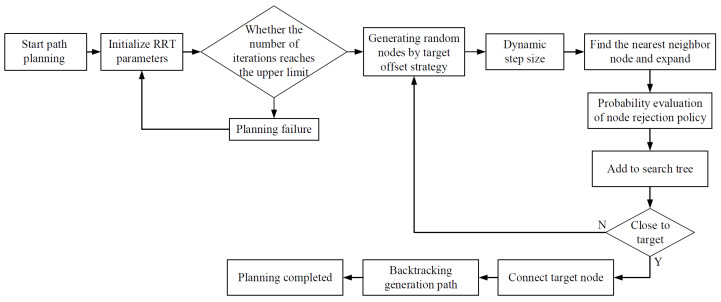
NDT-RRT path planning algorithm process.

Path planning for pickup is a crucial component for the precise and efficient operation of walnut harvesting robots. To improve the efficiency and quality of path planning, this paper proposes an improved NDT-RRT algorithm based on the Rapidly-exploring Random Tree (RRT) algorithm [28]. This algorithm quickly generates high-quality pickup paths based on target locations detected by the YOLO-FW model designed in this study. It significantly shortens planning time while maintaining excellent path quality. The entire path planning process is illustrated in Figure 7.

The path planning scenario in this study is conducted on a two-dimensional overhead view plane acquired by a monocular vision system. The planning space is defined by the robot’s current position (starting point), multiple target walnut pickup locations detected and localized by the YOLO-FW model, and obstacle areas formed by other non-target objects within the scene. The planning task is to find a continuous motion path for the robot in this two-dimensional unstructured orchard map. This path must start from the initial point, sequentially visit all target pickup points, and avoid obstacles. The optimization objective is to minimize both the path length and the planning time while ensuring collision-free operation.

#### 2.3.1. Node Rejection Strategy

During the random tree expansion process in the RRT algorithm, a large number of redundant nodes that contribute minimally to the final path are often generated. To address this issue, this study introduces a node rejection strategy [29], which eliminates invalid nodes in stages to improve path quality, reduce redundancy, and accelerate convergence. The strategy divides the planning process into three phases based on the maximum number of iterations: the exploration phase, the rejection phase, and the optimization phase. In the exploration phase, no node rejection occurs to allow for comprehensive exploration of the configuration space and avoid local optima. In the rejection phase, if the distance from a new node to the goal is greater than that from the nearest node to the goal, the new node is rejected with a probability of 1/2. The rejection probability is calculated as shown in Equations (17) and (18). In the optimization phase, both the node distance and path cost are considered to construct distance-based probability *P_distance_* and cost-based probability *P_cost_*, as detailed in Equations (19) and (20).

(17) $$P=\left\{\begin{array}{l} 0.5,\;\;d_{new-goal}>d_{near-goal}\\ 0,\;\;\;\;\;others \end{array}\right.$$

(18) $$d=\sqrt{{\left(x_{1}-x_{2}\right)}^{2}+{\left(y_{1}-y_{2}\right)}^{2}}$$

where *P* represents the probability of a node being rejected, *d_new-goal_* is the distance from the new node to the goal, *d_near-goal_* is the distance from the nearest node to the goal, and *d* is the Euclidean distance between the two points.

(19) $$P_{distance}=\left\{\begin{array}{l} 0.8,\;\;d_{new-goal}>d_{near-goal}\\ 0,\;\;\;\;\;others \end{array}\right.$$

(20) $$P_{cost}=\left\{\begin{array}{l} 0.5,\;\;C_{new}>C_{near}\\ 0,\;\;\;\;\;others \end{array}\right.$$

In the equations, *C_new_* and *C_near_* represent the cumulative path costs of the new and nearest nodes, respectively. In the optimization phase, if the new node is farther from the goal than the nearest node, it is rejected with an 80% probability. If its path cost exceeds 1.5 times that of the nearest node, it is rejected with a 50% probability. This design strengthens the dual screening of node quality in the path optimization phase, effectively excluding nodes that deviate from the goal or have excessively high costs, thereby guiding the generation of shorter, smoother feasible paths. The specific calculation of the path cost function is shown in Equation (21).

(21) $$C_{new}=C_{near}+\sqrt{{\left(x_{new}-x_{near}\right)}^{2}+{\left(y_{new}-y_{near}\right)}^{2}}$$

#### 2.3.2. Dynamic Step Length Strategy

**Figure 8:**
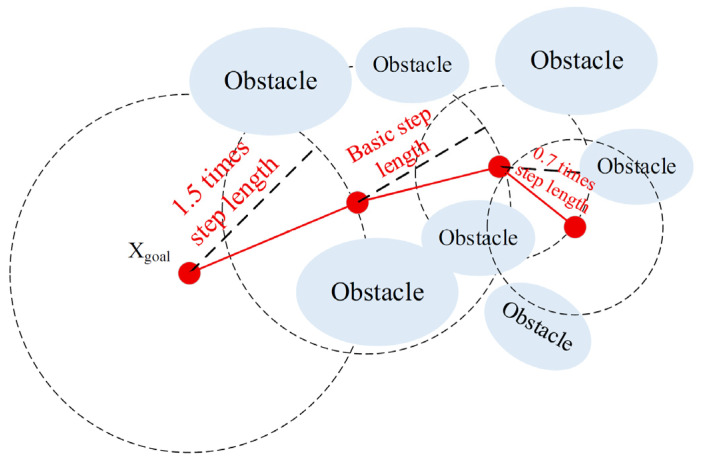
Schematic of the dynamic step length strategy.

In traditional RRT algorithms, the expansion step length of the random tree is usually fixed. A step length that is too large can lead to rough paths and difficulties in expanding in narrow areas, while a step length that is too small can slow down the search speed, particularly in open areas, causing slow convergence and negatively affecting the efficiency and practicality of path planning. To overcome these limitations, this paper introduces a dynamic step length strategy [30], allowing the step length to be adaptively adjusted based on the ratio of the current iteration count to the total iteration count. This provides the algorithm with the ability for “rapid exploration in the early stages and fine-tuned optimization in the later stages,” effectively balancing global search with local convergence, ultimately improving both path quality and search efficiency. The strategy is illustrated in Figure 8.

As shown in the schematic of the dynamic step length strategy, the step length varies across three stages with the iteration process: initial exploration phase, transition adjustment phase, and fine convergence phase. In the early stages of the algorithm (when the iteration count is less than one-third of the total iterations), the expansion step length is set to 1.5 times the base step length to facilitate rapid exploration of the configuration space and quickly locate feasible path regions. When the iteration count is between one-third and seven-tenths of the total iterations, the step length is linearly decayed according to Equation (22), gradually reducing from the base step length to 0.7 times its value, which maintains some exploration capability while gradually improving path precision and avoiding early convergence to local optima. In the final one-third of the iterations, the step length is kept at 0.7 times the base step length, focusing on local path optimization and smoothing, effectively avoiding obstacles and further improving path quality.

(22) $$S_{i}=l_{base}\times \left[\alpha -(\alpha -\beta )\times \frac{i}{i_{\max}}\right]$$

where *S_i_* represents the step length at the i-th iteration, *l_base_* is the base step length, α and β are the initial expansion and final reduction factors, respectively. *i_max_* is the maximum iteration count. In the experiments conducted in this paper, we set *l_base_* = 0.5, α=1.5, β=0.7.

#### 2.3.3. Target-Biased Sampling Strategy

Traditional RRT algorithms rely on random sampling across the entire space, which, while probabilistically complete, lack target-directed capabilities. This often results in the generation of redundant nodes and slow convergence. To address this, we employ a target-biased sampling strategy [31], which guides the growth of the search tree towards the goal by directly selecting the target point as a sample with a certain probability. This approach effectively limits the number of nodes and shortens the planning time.

In this strategy, a target bias probability *P* is set, and a random probability value *P*_1_ is uniformly generated within the interval [0, 1]. The sampling process is as follows: if $P_{1}<R$, the algorithm enters the goal-directed phase, directly selecting the goal point *x_goal_* as the sampling point, thereby expanding the new node towards the goal. If $R\leq P_{1}\leq P$, the process enters the target-biased sampling phase, where a sample point *x_sample_* is generated through a random sampling function within the configuration space. This process is mathematically expressed by Equations (23)–(25).

(23) $$x_{rand}=\left\{\begin{array}{l} x_{goal},\;P_{1}<R\\ x_{sample},\;R\leq P_{1}\leq P \end{array}\right.$$

(24) $$x=x_{nearest}+\lambda (x_{rand}-x_{nearest})$$

(25) $$y=y_{nearest}+\lambda (y_{rand}-y_{nearest})$$

In these equations, λ is the step size coefficient, *R* is the target sampling rate, which is set to 0.1 in this study.

To further enhance convergence efficiency, we incorporate dynamic adjustment by gradually decreasing the target bias probability *P* over the course of the iterations. The decay function is given by Equation (26). This design strengthens goal guidance in the early stages of the search, while gradually shifting back to random exploration in the later stages, balancing directionality and diversity, and thereby accelerating convergence towards a feasible path.

(26) $$P(i)=P_{basic}\times \varphi^{\frac{i}{i_{\max}}*k}$$

Here, *P_basic_* represents the initial bias probability, set to 0.3 in this experiment; φ is the decay coefficient, with a value of 0.99, and *k* is the decay speed adjustment factor, set to 10 here.

The settings of the initial bias probability, decay coefficient, and decay rate adjustment factor enable the algorithm to maintain a 30% target-oriented tendency in the early stage to accelerate convergence, while the gradual decay ensured by the decay coefficient prevents premature trapping in local optima. The adjustment factor *k* = 10 amplifies the decay effect, allowing the target bias to decrease progressively during the mid-phase, thereby increasing the probability of random exploration and enhancing search efficiency.

## 3. Results and Analysis

This study aims to explore the optimization of the picking path for detected targets based on a high-precision real-time detection model for Fallen Walnuts, using the performance metrics of both the detection model and path planning model for evaluation. All experiments were conducted on a Windows 11 system with the following configuration: AMD Ryzen Threadripper PRO3975WX 32-core processor, 3.50 GHz, 384 GB RAM, NVIDIA RTX A5000 GPU with 24 GB VRAM. The development environment included Python 3.8, CUDA 11.7, and Pytorch 1.13.

During the training phase, the stochastic gradient descent (SGD) optimizer was employed for iterative optimization. The number of epochs was set to 300, with a batch size of 16, a momentum factor of 0.937, an initial learning rate of 0.01, and a weight decay coefficient of 0.0005. Input images were resized to a resolution of 640 × 640 pixels to improve model performance. To increase dataset diversity and enhance model robustness, Mosaic data augmentation was applied throughout the training process.

### 3.1. Fallen Walnuts Detection Model Performance Analysis

To comprehensively evaluate the overall performance of the proposed YOLO-FW model, this study conducted a series of comparative experiments, including ablation studies, baseline model comparisons, and multi-scenario generalization analysis. The model’s performance was thoroughly assessed from multiple dimensions, including detection accuracy, robustness, and real-time performance.

#### 3.1.1. Ablation Study

To validate the effectiveness of the three proposed improvements, YOLOv11n was used as the baseline model. Ablation experiments were conducted by sequentially introducing each improvement method on a custom-built dataset, with the results shown in Table 1.

The results show that after introducing the StarNet module into the backbone network, the model’s memory usage and parameter count were reduced by 3.63% and 4.25%, respectively. Although detection accuracy and recall slightly decreased, they remained at 95.3% and 88.5%, respectively. This indicates that the module significantly improved computational efficiency and model lightweighting while maintaining the detection performance, making it more suitable for deployment on low-computing devices. Building on this, an improved CA_HSFPN feature fusion module was introduced into the neck network. This module increased mAP@50 by 0.4 percentage points to 95.7%, and recall was improved to 90.0%. While enhancing detection accuracy, the number of parameters, computational load, and memory usage were significantly reduced by 29.44%, 12.30%, and 28.03%, respectively. These results demonstrate that the introduced multi-scale feature selection and fusion mechanism effectively reduces computational resource consumption while ensuring detection performance. Finally, the introduction of the Inner_PIoU loss function further optimized the model. With no change in computational load, precision and recall improved by 0.2 and 0.4 percentage points, respectively.

Compared to the baseline model, the final YOLO-FW model improved recall and mAP@50 by 0.8 and 0.3 percentage points, respectively, while reducing the model’s parameter count, floating-point operations, and memory usage by 32.43%, 10.93%, and 30.65%, respectively. This shows the comprehensive advantages of the proposed improvements in both accuracy enhancement and model lightweighting.

#### 3.1.2. Comparison with Mainstream Baseline Models

To fully evaluate the comprehensive performance of the YOLO-FW model in the fallen walnuts detection task, this study compared it with the YOLOv5 to YOLOv12 series as well as other mainstream detection models such as Fast R-CNN and SSD. The results are shown in Table 2.

The experimental data indicate that YOLO-FW excels in model lightweighting, with its parameter count and memory usage being only 1.75 M and 3.62 MB, respectively. This represents a 32.43% and 30.65% decrease compared to the YOLOv11 baseline model. Compared to the latest YOLOv12 model, YOLO-FW’s parameter count and memory usage are also reduced by 30.56% and 30.12%, respectively, highlighting its significant lightweighting advantage. In terms of detection performance, the mAP@50 of YOLO-FW is only 0.1 and 0.4 percentage points lower than YOLOv12 and YOLOv9, respectively, while its mAP@50:95 reached 79.95%, achieving the highest value among all models. Its precision and recall rates are 90.6% and 90.4%, respectively, slightly lower than some models in the YOLO series, but still at a high level, fully meeting the detection requirements for fallen walnuts in practical orchard environments. In conclusion, YOLO-FW maintains competitive detection performance while offering excellent lightweight characteristics, making it more suitable for deployment in embedded devices with limited computational power.

#### 3.1.3. Detection Performance Analysis Across Multiple Scenarios

**Figure 9:**
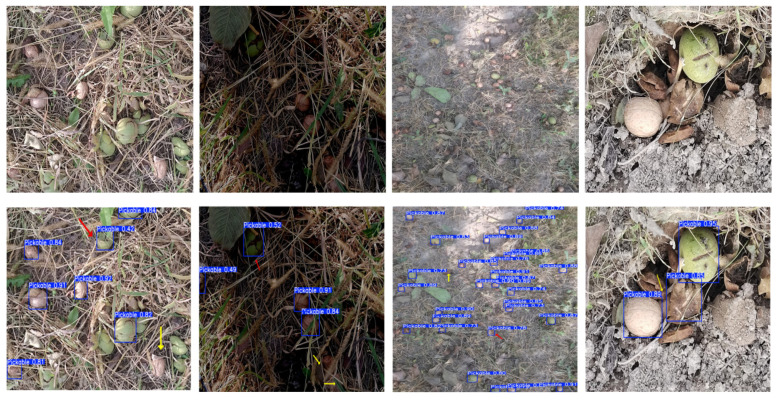

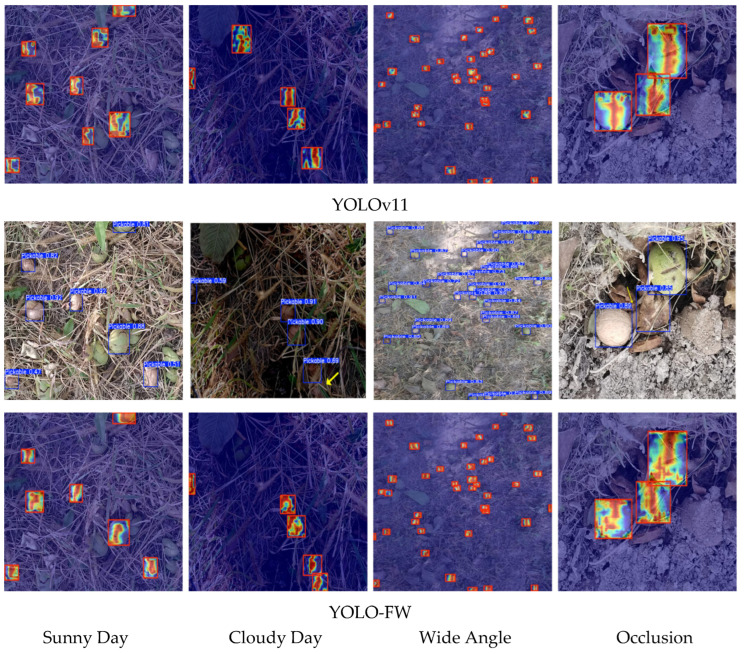
Comparison of detection performance across multiple scenarios.

To evaluate the detection performance of the YOLO-FW model in complex orchard environments, this study randomly selected data from the test set under various scenarios, including sunny days, cloudy days, wide-angle views, and occlusion. A comparative analysis was performed between the baseline model YOLOv11n and the improved YOLO-FW model. Some of the visualized detection results are shown in Figure 9.

In the figure, false positive detections are marked with red arrows, while missed detections are indicated with yellow arrows. It can be observed that in both sunny and wide-angle scenarios, YOLOv11n exhibits some false positives and missed detections. In contrast, the YOLO-FW model successfully detects all targets, demonstrating stronger environmental adaptability and robustness. Under cloudy conditions, YOLOv11n also shows significant false and missed detections, leading to reduced detection reliability. In comparison, YOLO-FW remains stable in recognizing most targets, achieving higher detection completeness. However, in scenarios with severe occlusion, neither model achieves complete detection, indicating that the current algorithm still has limitations in handling heavy occlusions. This remains an area for potential improvement in future work.

From the heatmap visualization results, it is clear that YOLO-FW focuses on a broader and more concentrated feature area, allowing it to more effectively hone in on key regions of the image related to fallen walnuts, thus enhancing its ability to distinguish useful features. In contrast, YOLOv11n exhibits a more dispersed heatmap, relying more on shallow features such as edges, which results in insufficient overall target representation in complex backgrounds, leading to more false and missed detections.

In summary, while YOLO-FW significantly reduces model parameters and memory usage, it maintains superior detection stability and generalization capability. It especially demonstrates stronger robustness in complex environments, making it more suitable for practical orchard fallen walnuts detection applications.

### 3.2. Fallen Walnuts Path Planning Model Performance Analysis

Building upon the previously developed Fallen Walnuts detection model, this study further evaluates the overall performance of the proposed NDT-RRT algorithm in the context of picking path planning.

#### 3.2.1. Comparison of Different Path Planning Algorithms

To verify the superiority of the NDT-RRT algorithm, this study conducted a comparative experiment with various classic path planning algorithms, including RRT, RRT*, RRT-Connect, A*, Ant Colony Optimization (ACO), and Genetic Algorithm (GA). The results are presented in Table 3.

The experimental data shows that the proposed NDT-RRT algorithm excels in terms of path planning time, path length, and the number of nodes generated. Compared to the traditional RRT algorithm, this algorithm reduces the planning time, path length, and node count by 87.71%, 0.53%, and 4%, respectively. When compared to the optimized variants RRT* and RRT-Connect, the planning time is reduced by 85.50% and 84.93%, respectively, while the path length is further shortened to 1289.03 mm. In comparisons with traditional planning methods such as A*, ACO, and GA, the planning time of NDT-RRT is only 0.56%, 0.30%, and 0.32%, respectively, of the aforementioned algorithms, significantly improving real-time performance. Moreover, this algorithm generates only 24 nodes during the entire planning process, demonstrating extremely high search efficiency.

In summary, the NDT-RRT algorithm maintains a short path while significantly reducing planning time and computational resource consumption, demonstrating excellent real-time performance and making it well-suited for deployment on embedded, low-computing platforms. Together with the previously proposed lightweight YOLO-FW detection model, this creates an efficient and practical detection-planning collaborative system for Fallen Walnuts picking robots.

#### 3.2.2. Path Planning Performance Comparison Across Multiple Scenarios

To comprehensively assess the performance of the proposed NDT-RRT algorithm in complex orchard environments, tests were conducted in both small-angle(target-concentrated) and wide-angle(target-dispersed) scenarios. These results were compared with several mainstream path planning algorithms, as shown in Table 4 and Table 5.

In the small-angle scenario, the NDT-RRT algorithm significantly outperforms the baseline RRT algorithm, reducing the path planning time by 46.21%, down to 0.78 s, with path length also being optimized. When compared to RRT, RRT-Connect, A*, ACO, and GA algorithms, the planning time was reduced by 64.71%, 62.32%, 99.14%, 99.78%, and 99.63%, respectively, with the path length increasing by only 0.5% compared to RRT-Connect. The results indicate that in relatively simple picking environments, the NDT-RRT algorithm has a significant advantage in planning efficiency, with a time performance improvement of over 45%.

**Figure 10:**
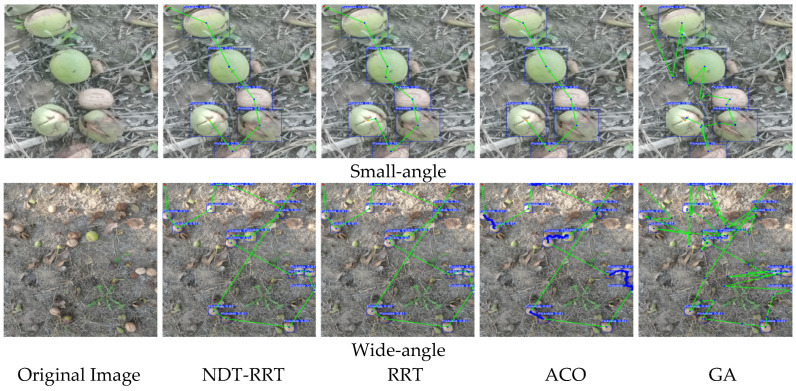
Path planning performance across multiple scenarios.

In the wide-angle, more complex scenario, NDT-RRT again demonstrated exceptional real-time performance, reducing planning time by 95.17% compared to RRT, with the path length remaining largely unchanged. Compared to other algorithms, the planning time was also shortened by more than 95%. Although the path length was slightly longer than that of RRT-Connect and A*, it remained within a reasonable range, and the path quality was competitive. A visualization of the path planning results for each algorithm can be seen in Figure 10.

Overall, the NDT-RRT algorithm maintains high planning efficiency and stability across different perspectives, especially in complex scenarios, without exhibiting significant performance fluctuations. In contrast, traditional algorithms generally suffer from longer planning times and decreased adaptability. This algorithm significantly improves search efficiency and scene adaptability while ensuring that path quality remains controllable. It is especially well-suited for deployment in embedded devices with limited computational power, meeting the dual requirements of real-time and stable path planning for orchard robots.

### 3.3. Model Deployment and Experimental Validation

**Figure 11:**
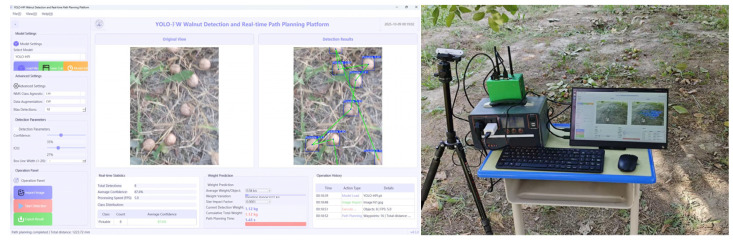
System integration verification.

To validate the comprehensive performance of the YOLO-FW detection model and NDT-RRT path planning algorithm in a real orchard environment, this study developed an integrated verification platform, as shown in Figure 11. The aforementioned model and algorithm were deployed on the NVIDIA Jetson Xavier NX (16 GB) embedded device, which features 384 CUDA cores and 48 Tensor cores, and utilizes the TensorRT inference framework for acceleration. This paper employs a Deep Learning Accelerator (DLA) to accelerate inference, utilizes FP16 mixed-precision inference to improve inference speed and reduce memory footprint, automatically selects optimal kernels to enhance parallel computational efficiency, and leverages a dynamic memory allocation mechanism to minimize latency.

The deployment test results indicated that the improved YOLO-FW model achieved a detection frame rate of 37.2 FPS on the edge device, representing a 50.6% increase compared to the original YOLOv11n model’s 24.7 FPS. Meanwhile, the NDT-RRT algorithm reduced the path planning time from 8.95 s to 4.93 s, improving efficiency by 44.92%. The experimental validation demonstrates that the proposed approach effectively meets the real-time object detection and rapid path planning requirements for orchard robots on low-power embedded devices, offering promising engineering applicability.

## 4. Discussion

This study presents a path planning method integrating star-shaped lightweight convolution and NDT-RRT, designed to address the slow decision-making response and low detection accuracy of agricultural robots in complex orchard environments. At the perception level, by introducing the star-shaped convolution (Star Blocks) to optimize the feature extraction capability of the backbone network, employing a multi-level scale pyramid feature network (CA_HSFPN) to enhance multi-scale feature fusion, and improving the CIoU loss function with the Inner-PIoU mechanism, the lightweight detection model YOLO-FW was developed.

Experiments demonstrate that the model exhibits good robustness and detection accuracy under complex lighting and multi-object scenarios. However, limitations in perception persist under severe occlusion, and future research will explore multi-modal spatial information fusion to enhance recognition performance under occlusion conditions.

At the path planning level, this study proposes the NDT-RRT algorithm based on the Rapidly-exploring Random Tree (RRT) algorithm, incorporating node rejection strategies, dynamic step-length mechanisms, and target-bias sampling strategies. The algorithm significantly improves search efficiency and real-time performance in complex multi-target scenarios, but there remains room for improvement in path smoothness and global optimality.

Future research will introduce path optimization mechanisms and extend the framework into a three-dimensional planning system, incorporating 3D environmental modeling to comprehensively enhance the operational capability and system applicability of picking robots in unstructured orchards.

## 5. Conclusions

(1) To address low recognition accuracy and insufficient real-time performance in the visual perception system of fallen walnut picking robots in complex orchard environments, this study proposes a YOLO-FW detection model based on star-shaped lightweight convolution. This model significantly reduces computational and storage overhead while maintaining high detection performance. The model’s parameter count and memory usage are only 1.75 M and 3.62 MB, representing a 32.43% and 30.65% decrease, respectively, compared to the YOLOv11n baseline model. When compared to the current mainstream YOLOv12 model, these reductions are 30.56% and 30.12%. Test results across various scenarios show that YOLO-FW retains good detection stability, generalization ability, and environmental robustness, while significantly compressing model complexity. This makes it well suited for real-time detection of fallen walnuts in actual orchard environments.

(2) To address the issues of low path planning efficiency and unreasonable trajectories in robotic arm grasping, this study builds upon the YOLO-FW detection results and proposes an improved NDT-RRT algorithm by integrating multiple strategies with the traditional RRT algorithm. By introducing node rejection strategies, dynamic step-size adjustment mechanisms, and target bias sampling methods, the algorithm reduces planning time, path length, and node count by 87.71%, 0.53%, and 4%, respectively, compared to the traditional RRT algorithm. Compared to advanced variants such as RRT* and RRT-Connect, NDT-RRT further reduces planning time by 85.50% and 84.93%, respectively, while optimizing the path length to 1289.03 mm. In comparison to traditional planning methods such as A*, ant colony optimization, and genetic algorithms, the planning time of NDT-RRT is only 0.56%, 0.30%, and 0.32%, respectively, of the time taken by these algorithms, demonstrating excellent real-time performance. Furthermore, validation results in both large and small field-of-view scenarios indicate that NDT-RRT maintains superior adaptability and planning efficiency in complex environments.

(3) To comprehensively evaluate the performance of the proposed algorithms in practical scenarios, an integrated detection and planning visualization system was developed based on the PySide6 framework and tested in a real orchard environment. The results show that the YOLO-FW model achieves an inference speed of 37.2 FPS on edge devices, a 50.6% improvement over the original YOLOv11 model. The NDT-RRT algorithm reduces path planning time from 8.95 s to 4.93 s, improving efficiency by 44.92%. The overall system testing validates the feasibility and comprehensive performance advantages of the proposed visual-planning integrated framework for fallen walnut picking robots in real-world complex orchard environments.

## Figures and Tables

**Table 1:** Ablation experiment results.

Model	StarNet	CA_HSFPN	Iner_PIoU	mAP@0.5/%	P/%	R/%	Params /M	FLOPs /G	Weights /MB
YOLOv11	×	×	×	95.4	91.9	88.6	2.59	6.4	5.22
	√	×	×	95.3	91.1	88.5	2.48	6.5	5.03
	√	√	×	95.7	90.6	90	1.75	5.7	3.62
	√	√	√	95.7	90.8	90.4	1.75	5.7	3.62

**Table 2:** Baseline model performance comparison.

Model	P/%	R/%	mAP@0.5/%	mAP0.5–0.95/%	Params/M	FLOPs/G	Weights/MB
SSD	62.7	47.7	62.72	30.82	23.7	273.2	90.6
Fast R-CNN	56.6	83.34	76.43	41.49	136.7	369.7	108
YOLOv5	91.2	90	95.7	78.68	1.92	4.2	3.9
YOLOv8	90.7	88.7	95.3	77.84	2.83	6.7	5.96
YOLOv9	92.6	90.8	96.1	79.42	70.5	315.6	142
YOLOv10	89.8	89.2	95.3	78.15	2.7	8.4	5.49
YOLOv11	91.9	88.6	95.4	78.89	2.59	6.4	5.22
YOLOv12	91.2	89.2	95.8	78.39	2.52	6	5.18
YOLO-FW	90.6	90.4	95.7	79.95	1.75	5.7	3.62

**Table 3:** Performance comparison results of different algorithms.

Algorithm	Average Path Planning Time (s)	Average Path Length (mm)	Average Number of Path Nodes
RRT	14.08	1295.8	25
RRT*	11.93	1289.89	25
RRT-connect	11.48	1291.17	24.6
A*	308.06	1290.15	24
ACO	568.07	1896.06	1302
GA	537.87	1996.99	49
NDT-RRT	1.73	1289.03	24

**Table 4:** Small-angle path planning test results.

Algorithm	Average Path Planning Time (s)	Average Path Length (mm)	Average Number of Path Nodes
RRT	1.45	649.97	13
RRT*	2.21	651.33	14.1
RRT-connect	2.07	651.33	12
A*	90.43	643.91	12
ACO	361	643.28	12
GA	211.79	933.90	24
NDT-RRT	0.78	643.38	12

**Table 5:** Wide-angle path planning test results.

Algorithm	Average Path Planning Time (s)	Average Path Length (mm)	Average Number of Path Nodes
RRT	47.5	1647.93	38
RRT*	92.5	1652.47	39.4
RRT-connect	46.22	1647.93	38
A*	134.53	1648.37	38
ACO	687.8	2189.29	1396
GA	1317.8	4360.04	78
NDT-RRT	2.29	1648.81	39

## Data Availability

The data that support the findings of this study are available within the manuscript.
